# Supplementary figures and images for: Multisystemic inflammatory disease in Pheasantshell (Unionidae, Actinonaias pectorosa) associated with Yokenella regensburgei infection at sites experiencing seasonal mass mortality events
Source: PLoS One. 2024 Aug 27;19(8):e0301250. doi: 10.1371/journal.pone.0301250 (PMC11349219; doi:10.1371/journal.pone.0301250)

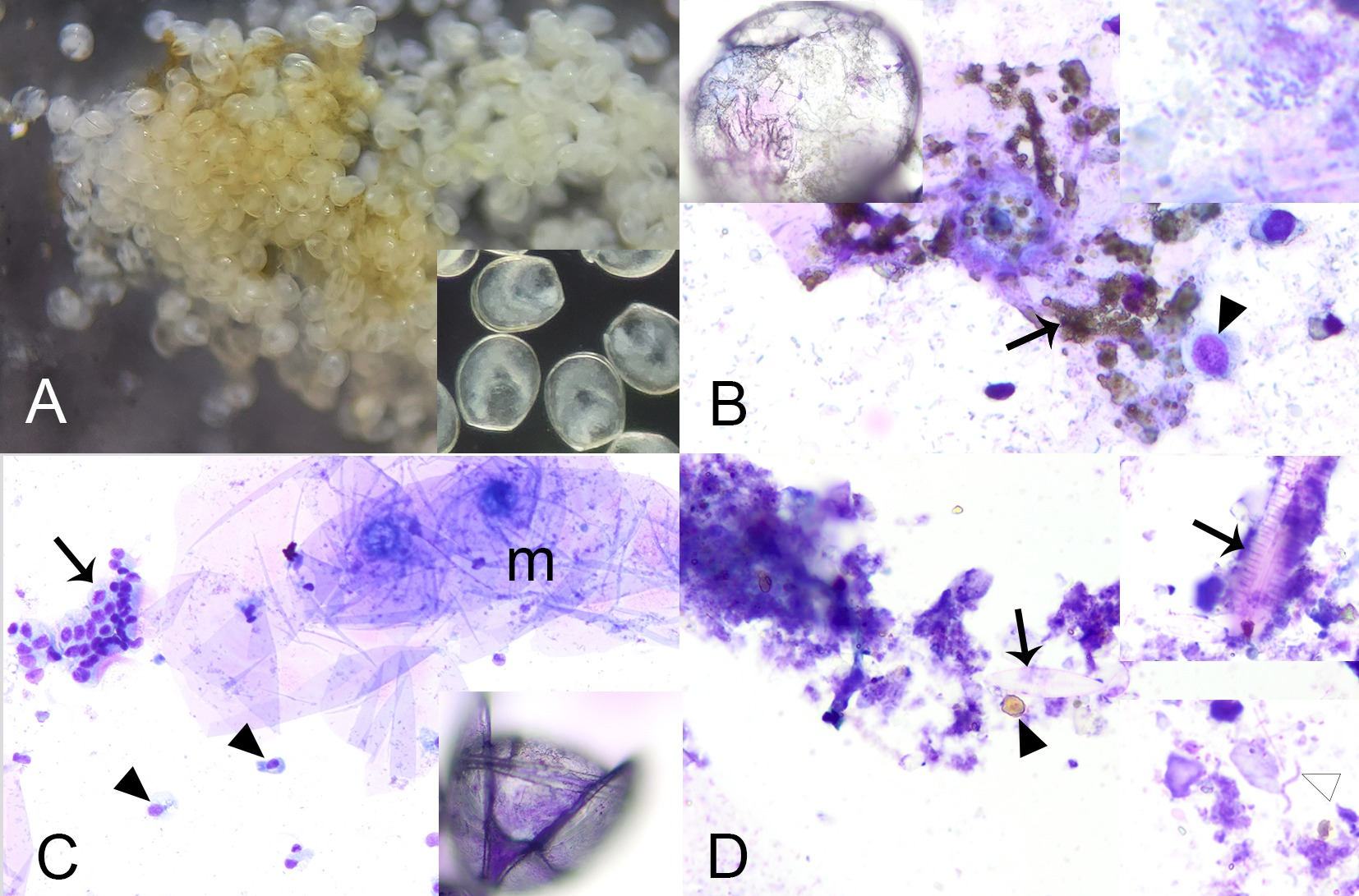

Supplement: S1 Fig — Cytological examination of tissues of case mussels (i.e., wild moribund Pheasantshell (Actinonaias pectorosa)) sampled at sites impacted by seasonal mortality events in the Clinch River. A. Wet-mounted impression of discolored marsupium with brown-tinged mucus and glochidia having poorly defined tissues (inset). B. Impression smear of discolored marsupium with abundant brown-granules (arrows) and agranular hemocytes (arrowhead) within a background of mucus containing abundant bacilli (inset right) and glochidia with poorly discerned tissues (inset left), modified Wright-Giemsa stain. C. Impression smear of normal-colored area of the same marsupium as B, with comparatively more mucus (m), intact ciliated epithelium (arrow) and hemocytes (arrowhead), and glochidia with discernable inner tissues (inset), modified Wright-Giemsa stain. D. Smear of fluid within the chamber of an anomalous shell consisting of amorphous acellular and crystalline debris, with scattered brown granules (black arrowhead), diatoms (arrow, inset upper), and cyanobacteria (white arrowhead, inset lower), modified Wright-Giemsa stain. (TIF) [file pone.0301250.s001.tif]
