# Supplementary material for: Multisystemic inflammatory disease in Pheasantshell (Unionidae, Actinonaias pectorosa) associated with Yokenella regensburgei infection at sites experiencing seasonal mass mortality events
Source: PLoS One. 2024 Aug 27;19(8):e0301250. doi: 10.1371/journal.pone.0301250 (PMC11349219; doi:10.1371/journal.pone.0301250)
Supplement: S1 Table — Total number of Pheasantshell (Actinonaias pectorosa) sampled by clinical presentation (case vs control) from two sites in Virginia (Speers Ferry and Sycamore Island) and one site in Tennessee (Kyles Ford). (DOCX) [file pone.0301250.s002.docx]

S1 Table

|  |  | Speers Ferry | Sycamore Island | Kyles Ford | Total |
| --- | --- | --- | --- | --- | --- |
| Case ^a^ | 2021 | 3 | 14 | 0 | 17 |
|  | 2022 | - | 4 | 0 | 4 |
|  | Total | 3 | 18 | 0 | 21 |
| Control ^b^ | 2021 | - | 24 | 24 | 48 |
|  | 2022 | - | 16 | 4 | 20 |
|  | 2023 | - | - | - | - |
|  | Total | - | 40 | 28 | 68 |

^a^ wild moribund Pheasantshell displaying clinical signs of disease

^b^ hatchery raised Pheasantshell kept in silos
