# Supplementary material for: Multisystemic inflammatory disease in Pheasantshell (Unionidae, Actinonaias pectorosa) associated with Yokenella regensburgei infection at sites experiencing seasonal mass mortality events
Source: PLoS One. 2024 Aug 27;19(8):e0301250. doi: 10.1371/journal.pone.0301250 (PMC11349219; doi:10.1371/journal.pone.0301250)
Supplement: S2 Table — Pheasantshell (Actinonais pectorosa) sampled by sex and clinical presentation from two sites in Virginia (Speers Ferry and Sycamore Island) and one site in Tennessee (Kyles Ford). (DOCX) [file pone.0301250.s003.docx]

S2 Table

|  | Male | Female | Immature | Hermaphrodite | Undetermined ^a^ | Total |
| --- | --- | --- | --- | --- | --- | --- |
| Case | 10 | 9 | 0 | 2 | 0 | 21 |
| Control | 13 | 1 | 35 | 0 | 19 | 68 |

^a^ gonad not histologically evaluated
