# Supplementary material for: Multisystemic inflammatory disease in Pheasantshell (Unionidae, Actinonaias pectorosa) associated with Yokenella regensburgei infection at sites experiencing seasonal mass mortality events
Source: PLoS One. 2024 Aug 27;19(8):e0301250. doi: 10.1371/journal.pone.0301250 (PMC11349219; doi:10.1371/journal.pone.0301250)
Supplement: S3 Table — Total number of Pheasantshell with trematode larvae per clinical presentation (case and control) identified in Pheasantshell (Actinonaias pectorosa) sampled from 2021–2023 across from two sites in Virginia (Speers Ferry and Sycamore Island) and one site in Tennessee (Kyles Ford), and total trematode count per site. We also identified trematode life stage and hemocyte response per clinical presentation across all three sites. (DOCX) [file pone.0301250.s004.docx]

S3 Table

|  |  | Case | | | |  | Control | | |
| --- | --- | --- | --- | --- | --- | --- | --- | --- | --- |
|  | Site | Speers Ferry | Sycamore Island | Kyles Ford | Total |  | Sycamore Island | Kyles Ford | Total |
|  | Sample size (n) | 3 | 18 | 0 | 21 |  | 40 | 28 | 68 |
|  | # of individuals with trematode larvae (%) | 0 | 9 (50) | - | 9 (43) |  | 11 (27) | 12 (43) | 23 (34) |
| Trematode life stage | metacercaria | - | 9 (100) | - | 9 (100) |  | 10 (91) | 12 (100) | 22 (96) |
|  | degenerate | - | 0 | - | 0 |  | 1 (9) | 0 | 1 (4) |
| Hemocyte response | none | - | 5 (56) | - | 5 (56) |  | 5 (46) | 3 (25) | 8 (35) |
|  | encapsulation | - | 1 (11) | - | 1 (11) |  | 1 (9) | 1 (8) | 2 (9) |
|  | nodulation | - | 3 (33) | - | 3 (33) |  | 4 (36) | 6 (50) | 10 (43) |
|  | focal hemocytic infiltration | - | 0 | - | 0 |  | 0 | 1 (8) | 1 (4) |
|  | multiple responses | - | 0 | - | 0 |  | 1 (9) | 1 (8) | 2 (9) |
