# Supplementary material for: Multisystemic inflammatory disease in Pheasantshell (Unionidae, Actinonaias pectorosa) associated with Yokenella regensburgei infection at sites experiencing seasonal mass mortality events
Source: PLoS One. 2024 Aug 27;19(8):e0301250. doi: 10.1371/journal.pone.0301250 (PMC11349219; doi:10.1371/journal.pone.0301250)
Supplement: S4 Table — Average relative abundance of indicator Operational Taxonomic Units (OTUs) per sample type and analysis-group. Samples consisted of hemolymph and digestive gland tissue collected from Pheasantshell (Actinonaias pectorosa) sampled from the Clinch River at two sites in Tennessee and Virginia. Analysis groups include Control-Absent (n = 62 DG and 6 HL), Case-Absent (n = 4 DG and 1 HL; individuals not presenting hemocytic nodulation and necrosis associated with gram-negative bacilli), and Case-Present (n = 11 DG and 6 HL; individuals presenting hemocytic nodulation and necrosis associated with gram-negative bacilli). Values in bold highlight the highest relative abundance per group. (DOCX) [file pone.0301250.s005.docx]

S4 Table

| OTU | Digestive Gland | | | Hemolymph | | |
| --- | --- | --- | --- | --- | --- | --- |
|  | Control-Absent | Case-Absent | Case-Present | Control-Absent | Case-Absent | Case-Present |
| Otu001_Rickettsiales | **45.2** | **20.0** | 0.3 | **30.2** | 5.0 | 0.3 |
| Otu002_Yokenella_sp | 0.2 | 4.0 | **37.1** | 0.2 | 4.0 | **52.8** |
| Otu003_Aeromonas_sp | 2.4 | 2.0 | 29.1 | 0.3 | 5.0 | 42.5 |
| Otu006_Bacteroides_sp | 0.0 | 0.3 | 8.3 | 0.0 | 0.0 | 0.3 |
| Otu008_Limnohabitans_sp | 0.1 | 1.0 | 0.5 | 12.7 | **37.0** | 0.0 |
| Otu010_Facklamia_sp | 3.0 | 6.3 | 0.0 | 14.3 | 0.0 | 0.0 |
| Otu023_Polynucleobacter_sp | 0.1 | 1.0 | 0.3 | 4.8 | 12.0 | 0.0 |
| Otu045_Bradyrhizobium_sp | 0.9 | 9.3 | 0.5 | 0.0 | 0.0 | 0.0 |
| Otu048_Hyphomicrobium_sp | 0.1 | 2.8 | 1.1 | 0.8 | 0.0 | 0.0 |
| Otu088_Bradyrhizobium_sp | 0.1 | 5.8 | 1.2 | 0.0 | 0.0 | 0.0 |
| Otu185_Legionella_sp | 0.0 | 1.0 | 0.0 | 0.0 | 0.0 | 0.0 |
| Otu Other | 47.8 | 46.8 | 21.6 | 36.7 | 37.0 | 4.0 |
